# Weight change across the start of three consecutive pregnancies and the risk of maternal morbidity and SGA birth at the second and third pregnancy

**DOI:** 10.1371/journal.pone.0179589

**Published:** 2017-06-19

**Authors:** Jacqueline M. Wallace, Sohinee Bhattacharya, Graham W. Horgan

**Affiliations:** 1 Rowett Institute, University of Aberdeen, Aberdeen, Scotland, United Kingdom; 2 Dugald Baird Centre for Research on Women’s Health, Aberdeen Maternity Hospital, Aberdeen, Scotland, United Kingdom; 3 Biomathematics & Statistics Scotland, Rowett Institute, University of Aberdeen, Aberdeen, Scotland, United Kingdom; Centro Cardiologico Monzino, ITALY

## Abstract

**Background:**

Weight-change across parities and/or current BMI may influence maternal and fetal morbidity and requires to be differentiated to better inform weight-management guidance.

**Methods:**

Direction, pattern and magnitude of weight-change across three consecutive parities and thereby two inter-pregnancy periods was described in 5079 women. The association between inter-pregnancy weight-change versus current BMI and adverse maternal events, SGA-birth and preterm delivery at second and third pregnancy were investigated by logistic regression.

**Results:**

More women gained weight across the defined childbearing period than lost it, with ~35% of normal and overweight women gaining sufficient weight to move up a BMI-category. Nine patterns of weight-change were defined across two inter-pregnancy periods and 50% of women remained weight-stable throughout (within 2BMI units/period). Women who were overweight/obese at first pregnancy had higher risk of substantial weight-gain and loss (>10kg) during each of two inter-pregnancy periods. Inter-pregnancy weight-gain (> 2BMI units) between first and second pregnancy increased the risk of maternal morbidity (1or more event of hypertensive disease, caesarean-section, thromboembolism) at second pregnancy, while weight-loss (>2BMI units) increased the risk of SGA-birth. Similarly, increased risk of maternal morbidity at the third pregnancy was influenced by weight-gain during both inter-pregnancy periods but not by current BMI-category. Both weight-gain between first and second pregnancy, and being overweight/obese by third pregnancy protected the fetus against SGA-birth whereas weight-loss between second and third pregnancy doubled the SGA risk.

**Conclusion:**

Half the women studied exhibited significant weight-fluctuations. This influenced their risk of maternal morbidity and SGA-birth at second and third pregnancy.

## Introduction

In an increasingly obesogenic environment the childbearing period is considered a key life-stage when women are vulnerable to excess weight-gain setting the scene for persistent weight-retention leading to mid-life obesity and its associated co-morbidities [1,2]. In spite of this assertion there is a relative paucity of longitudinal studies examining the direction and magnitude of weight-change between the start of successive parities in a defined population of women in relation to pre-gravid or early first pregnancy BMI. This information could be useful in identifying the categories of women most likely to benefit from interventions or public health advice aimed at mitigating the pregnancy-associated risk of excessive weight-gain. Thus our first objective was to describe the pattern of weight/BMI change between the first-ever, second and third pregnancies using weight and height measured in clinic during the first trimester, corrected to a standard stage of gestation and adjusted for important confounders such as age, year of delivery and inter-delivery interval.

In addition to the long-term implications of pregnancy-associated weight-gain, being overweight or obese, particularly at the outset of the first pregnancy, is associated with increased likelihood of experiencing pregnancy complications which impact the health and wellbeing of mother and baby. For mothers there is an increased risk of hypertensive disorders, gestational diabetes, thromboembolism, induction of labour and caesarean-delivery, while for the fetus there is higher risk of stillbirth, preterm delivery, macrosomia and fetal malformations such as spina bifida: both maternal and fetal risks generally increase with degree of overweight [3–6]. Maternal weight-change between pregnancies is also problematical and may occur during the baseline pregnancy and/or at some point after the baby is delivered and before the next successful conception. Irrespective of chronology, weight-gain between the first-trimester of the first-ever and second pregnancy robustly increases the risk of pre-eclampsia, gestational hypertension, gestational diabetes, caesarean-delivery, stillbirth, LGA (large-for-gestational age)-birth and infant mortality at the second pregnancy in geographically diverse populations [7–13]. For gestational hypertension, maternal diabetes, emergency caesarean-section, LGA-birth and infant mortality the risks associated with weight-gain are generally greater in women with a BMI below 25 units at first pregnancy [7,8,10,11,13]. Although the numbers involved are smaller, weight-loss between the first and second successful conception is also concerning as it is associated with an increased risk of spontaneous preterm delivery in women with a normal BMI at first pregnancy and with enhanced primary risk of low placental weight and SGA (small-for-gestational age)-birth irrespective of first pregnancy BMI [13,14].

To our knowledge the impact of inter-pregnancy weight-change on the risk of key maternal and fetal complications at the third pregnancy has not been documented. Thus our second objective was to investigate the association between weight-change during two inter-pregnancy periods spanning the first trimester booking appointment of three consecutive pregnancies on the risk of a composite maternal morbidity index, SGA-birth and spontaneous preterm delivery at the third pregnancy and compare this with current maternal BMI-category at the start of the third pregnancy.

## Methods

### Study population

The Aberdeen Maternity and Neonatal Databank (AMND) was the source of data for this retrospective cohort study, and the North of Scotland Research Ethics Service granted ethical approval (REC Ref 13/NS/0050) on the basis described previously [13]. Data were extracted for all singleton births after 24 weeks’ gestation in Aberdeen city and district from 1986–2013. The population was women who had their first-ever, second and third consecutive births (parity 0,1 and 2), who booked for antenatal care before 24weeks gestation on each occasion and whose weight and height were determined by trained clinical staff at the first antenatal (booking) visit. Women (n = 836) with missing data for variables of interest (mainly placental weight) were excluded resulting in a study population of 5,079 available for analysis.

### Study design

Maternal weight at the first booking visit for each of the three pregnancies was adjusted to take into account stage of gestation when weight was measured as described previously [13,15]. The resultant corrected weight was then used together with the unadjusted height to calculate an adjusted BMI (weight/height^2^). The mean individual difference in gestational age at the initial antenatal visit in the first versus the second, and second versus the third pregnancy was small (0.7±4.3 and 0.7±4.0 weeks, respectively, mean±sd) but this method ensured that the BMI calculated for all three pregnancies was corrected to the same stage of gestation for all women. Further as a number of women had their first antenatal visit after 16 weeks gestation (13.5, 9.0 and 6.1% in first, second and third pregnancies, respectively), this approach allowed us to include women who had a first booking appointment up to 24 weeks gestation.

The adjusted maternal BMIs were used to assign BMI-categories at the start of each pregnancy and to calculate the inter-pregnancy change in BMI between first and second, and second and third pregnancy. The former used standard cut-offs to define underweight (<18.5), normal weight (18.5–24.9), overweight (25.0–29.9) or obese (≥30), while for the latter women were grouped as follows: women who decreased their BMI by >2units (BMI/weight-loss), women who increased their BMI by >2units (BMI/weight-gain), and women who maintained their BMI within a 2unit loss or gain (BMI/weight-stable = reference) during each of two consecutive inter-pregnancy periods. Combining the categorisation for each period yielded nine groups that captured the pattern of weight-change in individual women over the defined childbearing period spanning all three pregnancies (namely gain-gain, gain-stable, gain-loss, stable-gain, stable-stable, stable-loss, loss-gain, loss-stable, loss-loss). The inter-delivery intervals between the birth of the first and second, and the second and third child were calculated in years.

Information on the pregnancy complications recorded in the AMND and known to robustly vary with BMI-status were used to derive a composite morbidity index (CMI) for adverse maternal events per pregnancy. These maternal events were pre-eclampsia and gestational hypertension (coded by ISSHP definition), emergency or elective Caesarean-section and thromboembolism. Individuals were characterised as having an adverse maternal event if they had at least one complication during the specified pregnancy. Gestational age was recorded according to the last menstrual period and confirmed by ultrasound. Spontaneous preterm delivery was defined as <37weeks gestation. Birthweight was defined as SGA if weight was <10^th^centile for gestation using gender and parity-specific birthweight charts for singleton births [16]. The placenta was weighed untrimmed and recorded to the nearest 10g.

### Statistical analysis

Maternal characteristics at three consecutive pregnancies in relation to first pregnancy (baseline) BMI-category were analysed by one-way ANOVA and where significantly different, categories were subsequently compared by Tukey’s method with significance levels at 1%. Pregnancy complication incidence at each pregnancy in relation to baseline BMI-category was analyzed by Chi-Square. The overall population characteristics at first versus second, and second versus third pregnancy were compared by paired Students-t-test for continuous variables and by McNemar’s test for categorical variables (Table 1). Average weight-change between the first and third pregnancy booking appointment in relation to BMI-category at first and third pregnancy was estimated by linear regression model (General Linear Model in Minitab) with adjustment for maternal age, height, year of delivery, booking week, smoking habit and inter-pregnancy interval as per the table footnote (Table 2). The crude and adjusted risk of substantial weight-gain or loss (defined as >10kg) in both inter-pregnancy periods in relation to whether women had a BMI below or above 25 at the first pregnancy was assessed using logistic regression with specific potential confounders adjusted for as per the table footnote (Table 3). A linear regression model was used to quantify weight-change across all three pregnancies in relation to the nine patterns of BMI-change observed as detailed above (Table 4). Finally, logistic regression was used to assess the risk of maternal morbidity, SGA-birth and spontaneous preterm delivery at the second or third maternity in relation to both inter-pregnancy BMI-change and current BMI-categories (Tables 5 and 6). Two regression models with and without adjustment for placental weight were used as we have previously shown that maternal BMI and inter-pregnancy BMI change impacts placental size [13]. Risks are presented as Odds Ratios (OR) with 95% confidence intervals (CI). McNemar’s test was performed using R (Foundation for Statistical Computing, Vienna, Austria) and all other analysis used Minitab (version 17; Minitab Inc., State College, PA).

**Table 1:** Maternal characteristics, birthweight, placental weight and the incidence of hypertensive disease, caesarean section, thromboembolism, spontaneous preterm delivery, and SGA-birth at first, second and third pregnancy in relation to BMI category at first pregnancy.

	Underweight	Normal	Overweight	Obese	P Value	All women
	BMI ≤ 18.5	BMI 18.6–24.9	BMI 25–29.9	BMI ≥30		
	n = 91	n = 3117	n = 1357	n = 514		n = 5079
First pregnancy						
Age (years)	21.8±4.32^b^	23.5±4.69^a^	24.5±4.75^c^	24.6±4.46^c^	<0.001	23.9±4.71
Height (cm)	162.5±6.51	162.3±6.32	162.2±6.35	162.2±6.27	0.907	162.3±6.32
Weight (kg)	45.8±4.52^b^	57.4±5.91^a^	69.0±6.74^c^	86.98±11.78^d^	<0.001	63.3±11.84
BMI (kg/m^2^)	17.3±1.08^b^	21.8±1.60^a^	26.2±1.52^c^	33.0±3.64^d^	<0.001	24.0±4.11
Booking week	13.7±4.10^b^	12.3±3.93^a^	11.5±3.90^c^	10.9±3.63^c^	<0.001	12.0±3.94
Smoking habit	39 (47.5%)^b^	927 (32.0%)^a^	319 (25.3%)^c^	143 (29.0%)^ac^	<0.001	1428 (30.1%)
Adjusted weight (kg)	46.9±4.36^b^	59.0±6.09^a^	70.9±6.70^c^	89.2±11.94^d^	<0.001	65.1±12.08
Adjusted BMI (kg/m^2^)	17.7±0.84^b^	22.4±1.56^a^	26.9±1.41^c^	33.8±3.64^d^	<0.001	24.7±4.17
Pre-eclampsia	4 (4.4%)^ab^	129 (4.1%)^a^	75 (5.5%)^b^	65 (12.6%)^c^	<0.001	273 (5.4%)
Gestational hypertension	15 (16.5%)^ab^	533 (17.1%)^a^	308 (22.7%)^bc^	136 (26.4%)^c^	<0.001	992 (18.5%)
Caesarean section	6 (6.6%)^a^	420 (13.5%)^b^	262 (19.3%)^c^	135 (26.3%)^d^	<0.001	823 (16.2%)
Thromboembolism	0	7 (0.2%)	3 (0.2%)	2 (0.4%)	n/a	12 (0.2%)
Maternal CMI^β^	23 (25.3%)^a^	962 (30.9%)^a^	568 (41.8%)^b^	282 (54.8%)^c^	<0.001	1835 (36.1%)
Spontaneous delivery (<37wks)	7 (7.7%)	218 (7.0%)	85 (6.3%)	26 (5.5%)	0.262	336 (6.6%)
SGA birth	27 (29.7%)^a^	377 (12.1%)^b^	120 (8.8%)^c^	40 (7.8%)^c^	<0.001	564 (11.1%)
Placental weight (g)	561±123^a^	601±138^a^	633±148^b^	640±151^b^	<0.001	613±143
Birth weight, non SGA (g)	3068±594^a^	3362±535^b^	3471±539^c^	3470±618^c^	<0.001	3399±550
Birth weight, SGA (g)	2571±353	2548±484	2537±561561	2486±504	0.881	2543±497
Second pregnancy						
Age (years)	25.4±4.91^a^	26.6±4.64^a^	27.4±4.69^b^	27.4±4.55^b^	<0.001	26.9±4.67***
Weight (kg)	48.7±5.76^b^	59.4±7.54^a^	71.9±10.15^c^	90.4±14.72^d^	<0.001	65.7±13.67***
BMI (kg/m^2^)	18.4±1.67^b^	22.5±2.43^a^	27.3±3.14^c^	34.3±4.95^d^	<0.001	24.9±4.85***
Booking week	12.5±4.00^a^	11.4±3.65^a^	11.0±3.62^b^	10.5±3.26^b^	<0.001	11.2±3.62***
Smoking habit	34 (40.0%)^b^	852 (28.9%)^a^	293 (22.5%)^a^	130 (26.3%)^a^	<0.001	1309 (27.1%)**
Adjusted weight (kg)	50.3±6.14^b^	61.4±7.7^a^	74.1±10.31^c^	92.8±14.93^d^	<0.001	67.8±13.90***
Adjusted BMI (kg/m^2^)	19.1±1.77^b^	23.3±2.42^a^	28.1±3.14^c^	35.2±4.97^d^	<0.001	25.7±4.89***
Pre-eclampsia	1 (1.1%)^a^	42 (1.3%)^a^	15 (1.1%)^a^	30 (5.8%)^b^	<0.001	88 (1.7%)***
Gestational hypertension	6 (6.6%)^ab^	177 (5.7%)^a^	134 (9.9%)^b^	65 (12.6%)^b^	<0.001	382 (7.5%)***
Caesarean section	10 (11.0%)^ab^	366 (11.7%)^a^	227 (16.7%)^b^	135 (26.3%)^c^	<0.001	738 (14.5%)*
Thromboembolism	0	9 (0.3%)	6 (0.4%)	2 (0.4%)	n/a	17 (0.3%)
Maternal CMI^β^	15 (16.5%)	562 (18.0%)	355 (26.2%)	207 (40.3%)	<0.001	1139 (22.4%)***
Spontaneous delivery (<37wks)	11 (12.1%)^a^	212 (6.8%)^ab^	75 (5.5%)^b^	26 (5.1%)^b^	0.041	324 (6.4%)
SGA birth	14 (15.4%)^a^	326 (10.4%)^a^	94 (6.9%)^b^	33 (6.4%)^b^	<0.001	467 (9.2%)**
Placental weight (g)	588±113^b^	638±142^a^	668±145^c^	686±164^c^	<0.001	650±146*
Birth weight, non SGA (g)	3243±429^b^	3485±528^a^	3578±541^c^	3594±590^c^	<0.001	3518±540***
Birth weight, SGA (g)	2648±356^ab^	2698±377^a^	2671±562^a^	2294±803^b^	<0.001	2663±470***
Third pregnancy						
Age (years)	29.5±4.81^ab^	30.6±4.81^a^	31.1±4.81^b^	31.0±4.58^ab^	<0.001	30.8±4.81^¥¥¥^
Weight (kg)	50.8±7.08^b^	61.9±8.68^a^	74.8±11.84^c^	93.8±17.36^d^	<0.001	68.4±14.90^¥¥¥^
BMI (kg/m^2^)	19.2±2.17^b^	23.5±2.91^a^	28.4±3.90^c^	35.6±6.11^d^	<0.001	25.9±5.36^¥¥¥^
Booking week	11.0±3.63^ab^	10.7±3.38^a^	10.5±3.47^a^	9.9±3.14^b^	<0.001	10.5±3.39^¥¥¥^
Smoking habit	32 (36.8)^a^	819 (27.0%)^ac^	296 (22.4%)^bc^	127 (25.1%)^bc^	0.001	1274 (25.8%)
Adjusted weight (kg)	53.0±7.31^b^	64.2±8.92^a^	77.1±12.02^c^	96.4±17.58^d^	<0.001	70.7±15.12^¥¥¥^
Adjusted BMI (kg/m^2^)	20.0±2.23^b^	24.4±2.96^a^	29.3±3.93^c^	36.6±6.16^d^	<0.001	26.8±5.41^¥¥¥^
Pre-eclampsia	2 (2.2%)	25 (0.8%)^a^	21 (1.5%)^b^	17 (3.3%)^c^	<0.001	65 (1.3%)
Gestational hypertension	2 (2.2%)^a^	135 (4.3%)^b^	93 (6.8%)^c^	53 (10.3%)^c^	<0.001	283 (5.6%)^¥¥¥^
Caesarean section	13 (14.3%)^ab^	501 (16.1%)^a^	298 (22.0%)^b^	171 (33.3%)^c^	<0.001	983 (19.4%)^¥¥¥^
Thromboembolism	1 (1.1%)	12 (0.4%)	3 (0.2%)	4 (0.8%)	n/a	20 (0.4%)
Maternal CMI^β^	17 (18.7%)	649 (20.8%)	393 (29.0%)	224 (43.6%)	<0.001	1283 (25.3%)^¥¥^
Spontaneous delivery (<37wks)	12 (13.2%)^a^	197 (6.3%)^b^	56 (4.1%)^c^	35 (6.8%)^ab^	0.001	300 (5.9%)
SGA birth	11 (12.1%)^ab^	274 (8.8%)^a^	86 (6.3%)^b^	31(6.0%)^b^	0.005	402 (7.9%)^¥^
Placental weight (g)	604±140^b^	652±143^a^	681±147^c^	699±158^c^	<0.001	664±147
Birth weight, non SGA (g)	3174±554^b^	3522±512^a^	3617±514^c^	3653±567^c^	<0.001	3556±524
Birth weight, SGA (g)	2637±411	2628±415	2624±531	2554±538	0.861	2622±451
Inter-delivery interval (years)						
First and second	3.6±2.78	3.1±2.23	2.9±1.90	2.9±1.74	0.002	3.0±2.12
Second and third	4.2±2.68^ab^	4.0±2.80^a^	3.7±2.49^ab^	3.5±2.50^b^	<0.001	3.9±2.69
First and third	7.8±3.61^ab^	7.1±3.49^a^	6.6±3.10^bc^	6.4±2.93^c^	<0.001	6.9±3.35

Values are mean (SD) or number (per cent).

^β^CMI, combined morbidity index (see methods text). Univariate analysis was conducted using one-way ANOVA for continuous variables and where significant effects were detected, individual maternal BMI categories were subsequently compared by Tukey’s method. Chi-Square tests were used to test independence among all possible BMI group comparisons for categorical variables. Within rows any means which do not have a superscript in common are significantly different, and otherwise are not, minimum P<0.01. The overall population characteristics at first versus second pregnancy, and second versus third were compared by paired Students t test for continuous variables and by McNemar’s test for categorical variables. First versus second comparison significance

*P<0.05

**P<0.01

***P<0.001; second versus third

^¥^P<0.05

^¥¥^P<0.01

^¥¥¥^P<0.001.

**Table 2:** Average weight change (kg) between first and third pregnancy in relation to BMI classification at baseline (first) and third pregnancy. Within baseline BMI category columns percentage of women per BMI category at third pregnancy in square brackets.

	BMI category at first pregnancy (number of women)			
BMI category at third pregnancy	Underweight	Normal	Overweight	Obese
	n = 91	n = 3117	n = 1357	n = 514
Underweight	0.76±0.930 [23.1%]	-5.28±1.15 [0.4%]	n/a [0%]	n/a [0%]
Normal	7.19±0.510 [73.6%]	2.19±0.09 [64.2%]	-4.96±0.49 [10.7%]	-20.46±4.19 [1.2%]
Overweight	18.40±2.49 [3.3%]	9.06±0.13 [30.6%]	2.57±0.22 [52.3%]	-7.03±1.30 [12.0%]
Obese	n/a [0%]	21.77±0.33 [4.8%]	14.51±0.27 [36.9%]	9.53±0.47 [86.8%]

Maternal weight measured at the first and third pregnancy booking appointment was adjusted to a standard gestational age for all women as detailed previously [13,15].Weight change (fitted mean ± SEM) estimated by general linear model and adjusted for maternal age, height and year of delivery at first pregnancy, booking week and smoking habit at first and third pregnancy and inter- delivery interval between first and third pregnancy.

**Table 3:** Frequency rate and risk of substantial weight gain or loss (>10kg) in two consecutive inter-pregnancy periods for women with a BMI below or above 25 units at first pregnancy.

		>10kg weight gain between pregnancy 1 and 2	>10kg weight loss between pregnancy 1 and 2	>10kg weight gain between pregnancy 2 and 3	>10kg weight loss between pregnancy 2 and 3
BMI below 25 units at first pregnancy (n = 3208)	Rate (%)	7.23	0.18	8.91	0.46
	Crude OR (95% CI)	1	1	1	1
	Adjusted OR (95% CI)	1	1	1	1
BMI above 25 units at first pregnancy (n = 1871)	Rate (%)	16.83	2.89	14.64	2.93
	Crude OR (95% CI)	2.59 (2.16–3.11)***	15.86 (6.82–36.93)***	1.73 (1.46–2.09)***	6.44 (3.63–11.44)***
	Adjusted OR (95% CI)	2.53 (1.90–3.36)***	5.23 (2.01–13.62)***	1.69 (1.31–2.21)***	2.18 (1.05–4.50)*

Odds ratios (OR) and 95% confidence limits (CI) from logistic regression

*P<0.05

***P<0.001. BMI below 25 units at first pregnancy = reference group. For >10kg weight change between pregnancy 1 and 2, model adjusted for baseline BMI, height, age, and year of delivery at first pregnancy, inter-delivery interval between pregnancy 1 and 2, and booking week and smoking status at both these pregnancies. For >10kg weight change between pregnancy 2 and 3, model adjusted for baseline BMI, height, age, and year of delivery at second pregnancy, inter-delivery interval between pregnancy 2 and 3, and booking week and smoking status at both these pregnancies.

**Table 4:** Average weight and BMI change in relation to pattern of BMI-change between first, second and third pregnancy.

Pattern of BMI change between 3 pregnancies	Number of women (% of population)	Weight change–pregnancy 1 to 2	Weight change–pregnancy 2 to 3	BMI change–pregnancy 1 to 2	BMI change–pregnancy 2 to 3
Gain-gain	362 (7.1%)	10.37±0.196^b^	10.67±0.191^a^	3.96±0.073^b^	4.07±0.072^a^
Gain-stable	795 (15.6%)	10.00±0.133^b^	0.51±0.129^d^	3.79±0.050^b^	0.20±0.048^d^
Gain-loss	151 (2.9%)	11.97±0.30^a^	-9.13±0.291^e^	4.53±0.113^a^	-3.48±0.110^e^
Stable-gain	872 (17.2%)	0.44±0.128^c^	9.65±0.124^b^	0.17±0.048^c^	3.67±0.047^b^
Stable-stable	2513 (49.5%)	0.75±0.074^c^	1.28±0.072^c^	0.29±0.028^c^	0.49±0.027^c^
Stable-loss	116 (2.3%)	1.46±0.340^c^	-8.15±0.330^e^	0.55±0.128^c^	-3.07±0.125^e^
Loss-gain	125 (2.5%)	-9.29±0.328^e^	11.15±0.318^a^	-3.51±0.123^e^	4.25±0.121^a^
Loss-stable	135 (2.6%)	-7.59±0.315^d^	1.79±0.306^c^	-2.87±0.119^d^	0.67±0.116^c^
Loss-loss	10 (0.2%)	-8.49±1.160^de^	-9.04±1.12^e^	-3.18±0.435^de^	-3.39±0.426^e^
P value		<0.001	<0.001	<0.001	<0.001

Maternal weight measured at the first, second and third pregnancy booking appointment was adjusted to a standard gestational age for all women as detailed previously [13,15]. BMI change within each specified inter-pregnancy period defined as loss if BMI decreased by >2 units, gain if BMI increased by >2 units and stable if BMI remained within 2 units of the previous pregnancy value. Weight/BMI change (fitted mean ± SEM) was estimated by general linear model and adjusted for maternal age, height and year of delivery at first pregnancy, booking week and smoking habit at first and second or second and third pregnancy as appropriate and inter-delivery interval between first and second or second and third pregnancy as appropriate.Within columns, means with a different superscript differ at P<0.01 (Tukey pairwise comparisons).

**Table 5:** Frequency rate and adjusted risk of maternal morbidity, SGA-birth and spontaneous preterm delivery during second pregnancy in relation to change in BMI between first and second pregnancy, and BMI category at second pregnancy booking.

		Model 1- second pregnancy outcome		
		Maternal Morbidity	SGA-birth	Spontaneous Preterm <37weeks
Number of affected pregnancies	Rate (%)	1139 (22.4%)	467 (9.2%)	324 (6.4%)
Categorical predictors				
BMI gain vs. stable, Pregnancy 1–2	OR(95% CI)	1.32 (1.09–1.60)**	0.81 (0.60–1.11)	0.98 (0.65–1.48)
BMI loss vs. stable, Pregnancy 1–2	OR(95% CI)	1.14 (0.84–1.57)	1.88 (1.24–2.85)**	1.41 (0.78–2.57)
At Pregnancy 2				
Obese vs. normal	OR(95% CI)	1.38 (0.96–1.99)	1.18 (0.67–2.10)	0.82 (0.31–1.60)
Overweight vs. normal	OR(95% CI)	1.19 (0.97–1.45)	0.80 (0.58–1.09)	0.75 (0.48–1.15)
Undernourished vs. normal	OR(95% CI)	1.51 (0.76–2.99)	1.16 (0.57–2.36)	1.63 (0.70–3.82)
		Model 2 –second pregnancy outcome		
		Maternal Morbidity	SGA-birth	Spontaneous Preterm <37weeks
Number of affected pregnancies	Rate (%)	1139 (22.4%)	467 (9.2%)	324 (6.4%)
Categorical predictors				
BMI gain vs. stable, Pregnancy 1–2	OR(95% CI)	1.29 (1.06–1.56)**	0.86 (0.62–1.19)	0.96 (0.64–1.46)
BMI loss vs. stable, Pregnancy 1–2	OR(95% CI)	1.16 (0.84–1.59)	2.07 (1.32–3.22)***	1.41 (0.77–2.56)
At Pregnancy 2				
Obese vs. normal	OR(95% CI)	1.34 (0.93–1.94)	1.26 (0.68–2.34)	0.67 (0.30–1.50)
Overweight vs. normal	OR(95% CI)	1.16 (0.94–1.41)	0.86 (0.61–1.20)	0.72 (0.47–1.12)
Undernourished vs. normal	OR(95% CI)	1.63 (0.82–3.22)	0.94 (0.43–2.04)	1.72 (0.73–4.03)

Maternal morbidity if an individual had one or more complications including pre-eclampsia, gestational hypertension, caesarean delivery or thromboembolism during second pregnancy. Odds ratios and 95% confidence limits from logistic regression

*P<0.05

**P<0.01

***P<0.001. Model 1 adjusted for baseline BMI at first pregnancy, height, inter-delivery interval between first and second pregnancy, and maternal age, year of delivery, smoking habit, gestational age and baby gender at second pregnancy. Model 2 additionally adjusted for placental weight at second delivery.

**Table 6:** Frequency rate and adjusted risk of maternal morbidity, SGA-birth and spontaneous preterm delivery during third pregnancy in relation to change in BMI between three consecutive pregnancies and BMI category at third pregnancy booking.

	Model 1- third pregnancy outcome		
	Maternal Morbidity	SGA-birth	Spontaneous Preterm <37weeks
Number of affected pregnancies	1283 (25.3%)	402 (7.9%)	300 (5.9%)
Categorical predictors			
BMI gain vs. stable, Pregnancy 1–2	1.24 (1.04–1.49)*	0.69 (0.51–0.95)*	1.00 (0.67–1.51)
BMI loss vs. stable, Pregnancy 1–2	0.81 (0.59–1.11)	1.09 (0.68–1.72)	1.04 (0.54–2.01)
BMI gain vs. stable, Pregnancy 2–3	1.35 (1.12–1.61)***	1.15 (0.87–1.54)	0.69 (0.45–1.06)
BMI loss vs. stable, Pregnancy 2–3	0.88 (0.65–1.20)	2.23 (1.47–3.39)***	0.67 (0.34–1.35)
At Pregnancy 3			
Obese vs. normal	1.06 (0.77–1.44)	0.56 (0.33–0.94)*	0.97 (0.49–1.94)
Overweight vs. normal	1.07 (0.89–1.29)	0.58 (0.43–0.77)***	0.67 (0.43–1.02)
Undernourished vs. normal	0.58 (0.19–1.77)	1.13 (0.37–3.39)	2.76 (0.97–7.88)
	Model 2- third pregnancy outcome		
	Maternal Morbidity	SGA-birth	Spontaneous Preterm <37weeks
Number of affected pregnancies	1283 (25.3%)	402 (7.9%)	300 (5.9%)
Categorical predictors			
BMI gain vs. stable, Pregnancy 1–2	1.22 (1.01–1.46)*	0.69 (0.49–0.98)*	0.96 (0.66–1.49)
BMI loss vs. stable, Pregnancy 1–2	0.79 (0.58–1.09)	1.12 (0.68–1.84)	1.04 (0.54–2.01)
BMI gain vs. stable, Pregnancy 2–3	1.32 (1.01–1.58)**	1.25 (0.91–1.70)	0.70 (0.45–1.07)
BMI loss vs. stable, Pregnancy 2–3	0.92 (0.68–1.25)	2.18 (1.39–3.43)***	0.71 (0.36–1.42)
At Pregnancy 3			
Obese vs. normal	0.97 (0.70–1.32)	0.64 (0.36–1.14)	0.91 (0.45–1.82)
Overweight vs. normal	1.01 (0.84–1.22)	0.67 (0.49–0.93)*	0.63 (0.41–0.97)
Undernourished vs. normal	0.57 (0.19–1.76)	1.19 (0.36–3.93)	2.78 (0.97–7.91)

Maternal morbidity if women had one or more complications including pre-eclampsia, gestational hypertension, caesarean delivery or thromboembolism at third maternity. Odds ratios and 95% confidence limits from logistic regression

*P<0.05

**P<0.01

***P<0.001. Model 1 adjusted for baseline BMI at first pregnancy, height, inter-delivery interval between first and second pregnancy, and second and third pregnancy, and maternal age, year of delivery, smoking habit, gestational age and baby gender at third pregnancy. Model 2 additionally adjusted for placental weight.

## Results

### Maternal characteristics and weight-change across parities

Maternal characteristics at the first, second and third pregnancy are detailed in relation to the BMI-category at the first baseline pregnancy (Table 1). At the baseline pregnancy 1.8% of women were underweight, 61.4% normal weight, 26.7% overweight and 10.1% obese. Relative to women with a normal BMI at first pregnancy those categorized as obese were older and had an earlier booking appointment on all three occasions. There was no difference in the inter-delivery interval between the first and second pregnancy but initially obese women had the shortest interval between their second and third deliveries.

The percentage of women moving between BMI-categories and the magnitude of weight-change between the first and third pregnancy booking appointment are detailed in Table 2. The majority (73.6%) of initially underweight women gained enough weight (~7kg) to be categorized as normal BMI by the third pregnancy. Of women with a normal BMI initially, 35.4% had gained sufficient weight to reclassify them as overweight or obese by the start of the third pregnancy, corresponding to an average weight-gain of ~9 and 21kg, respectively. Similarly, 36.9% of overweight women gained an average of 14kg and were obese by the third pregnancy while in the initially obese group, 86% remained obese and had gained a further 9kg by the third pregnancy. In contrast a modest percentage of both overweight (10.7%) and obese women (13.2%) lost sufficient weight to move down at least one BMI-category between the first and third pregnancy. For the population overall, the frequency of substantial weight-gain between pairs of consecutive pregnancies was much more common than weight-loss (both defined as >10kg, Table 3) but the number of women that experienced substantial gain or loss between the first and second compared with the second and third pregnancies was equivalent (547 vs 560, and 60 vs 70, respectively). Furthermore the magnitude of this substantial weight-change, adjusted for confounders as per Table 3 (fitted mean ± SEM), was similar in both inter-pregnancy periods (gain, 14.9±0.19 and 14.4±0.20kg: loss 15.7±0.74 and 15.2±0.66kg, P>0.1). When examined by logistic regression women with a BMI above 25 units at the outset of childbearing were at increased risk of both substantial weight-loss and weight-gain between the first and second, and second and third pregnancy compared with women who had a BMI below 25 at baseline (Table 2).

The above analysis does not fully capture the direction and magnitude of weight/BMI-change in individual women: this is detailed for nine possible patterns in Table 4. Almost 50% of the population remained weight-stable in both consecutive inter-pregnancy periods. The second most numerous pattern was women who were weight-stable between first and second pregnancy and gained weight between the second and third (17.2%), closely followed by the group that gained between first and second pregnancy and were weight-stable thereafter (15.6%). Unsurprisingly the least prevalent pattern was in 10 women (0.2%) who lost weight during both inter-pregnancy periods. The extent of average weight-loss between the second and third pregnancy was equivalent in the gain-loss, stable-loss and loss-loss groups. For those who gained between the second and third pregnancy, the increase in weight was similar in the gain-gain and loss-gain groups and slightly greatly than in the stable-gain group but the average difference was only 1–1.5kg. For the women who remained weight-stable between the second and third pregnancy, the average weight-change was gain-stable<stable-stable and loss-stable and again the differential between stable groups were small. Overall, there was no difference in the number of women who gained or lost >2 BMI units between the first and second pregnancy compared with the second and third pregnancy (1308 vs.1359, P>0.25 and 270 vs. 277, P>0.758, respectively). Moreover the magnitude of weight-change, adjusted for confounders as per Table 4, was equivalent in both inter-pregnancy periods (gain, 10.3±0.11 and 10.1±0.10kg: loss, 8.4±0.22 and 8.7±0.22kg, P>0.1).

### Pregnancy complications, inter-pregnancy BMI-change and current BMI

Pregnancy complication incidence at all three pregnancies are detailed in relation to BMI-category at first baseline pregnancy (Table 1). At the first pregnancy obese women had the highest crude incidence of hypertensive disease, caesarean-section and thromboembolism, and the lowest incidence of SGA-birth. Accordingly the combined morbidity index for maternal events at first pregnancy was obese>overweight>normal and underweight. For the population as a whole the frequency of pre-eclampsia, gestational hypertension, and SGA-birth decreased with increasing parity while the incidence of caesarean-delivery increased, predominantly between the second and third pregnancy. The incidence of spontaneous preterm delivery (<37 weeks) was independent of parity. The risk of maternal morbidity, SGA-birth and spontaneous preterm delivery at the second and third pregnancy were examined in relation to inter-pregnancy weight-change(s) and current BMI-categories. For adverse events during the second pregnancy, BMI-category at booking was without influence (Table 5). In contrast inter-pregnancy weight/BMI-gain between first and second pregnancy booking increased the adjusted risk of maternal morbidity, while weight-loss during the same period increased the risk of SGA-birth. These risks remain after adjustment for placental weight at the second delivery.

For adverse events at the third pregnancy (Table 6), inter-pregnancy weight/BMI-gain between both the first and second, and second and third pregnancies contributed to the increased adjusted risk of maternal morbidity at the third pregnancy. In contrast, maternal BMI category at the third pregnancy booking did not influence the risk of maternal complications in any way. Both weight-gain between first and second pregnancy, and being overweight or obese by third pregnancy booking protected the fetus against SGA-birth whereas weight-loss between second and third pregnancy doubled the risk of SGA-birth. These relationships remained after additional adjustment for placental weight at the third delivery with one exception: the protective effect of maternal obesity on the risk of SGA-birth was no longer significant. Inter-pregnancy weight/BMI-change and current pregnancy BMI-category did not influence the risk of spontaneous preterm delivery at second or third pregnancy.

## Discussion

### Weight-change across the start of three consecutive pregnancies

This retrospective analysis of maternal weight-change during a defined period of childbearing spanning the start of three pregnancies reveals that half the women studied exhibited significant fluctuations in weight in one or both inter-pregnancy periods. Unsurprisingly weight-gain was more common than weight-loss and a third of women with a normal BMI at baseline gained sufficient weight to be re-classified as overweight or obese by the beginning of the third pregnancy. For these women the magnitude of weight-gain over an average period of 8 years was considerable (~11kg) and would if retained theoretically impact health in mid-life and beyond. Accordingly public health advice aimed at limiting pregnancy-associated weight-gain needs to be aware that women who start their childbearing years at a healthy weight are not completely without risk. With high rates of obesity in the obstetric population [17], the focus understandably leans towards policies to limit further weight-gain in women who are initially overweight or obese. Indeed our analysis confirms the perception that women with a BMI above 25 units at the baseline pregnancy are at higher risk of substantial weight-gain (here defined as >10kg) between both the first and second, and second and third pregnancies. However a proportion of the initially overweight/obese women are also at increased risk of substantial weight-loss between successive parities. In support recent studies report wider variability in gestational weight-gain in overweight/obese women and while ~70% of obese women exceed current pregnancy weight-gain recommendations, gestational weight-loss is also more common in this group, and increasingly prevalent as the severity of obesity increases [18,19]. Greater variability in weight-change between parities has also been reported for women who had a high BMI or were heavier than normal at the first baseline pregnancy [13,20]. The underlying causes of greater fluctuations in inter-pregnancy weight in overweight/obese women are beyond the scope of this study but may reflect a mixture of lifestyle choices such as dietary-restraint, smoking, choosing to breastfeed, exercise level or biological factors as indicated by rates of vomiting while pregnant, short sleep duration post-partum, age at menarche and at first birth [21–26].

USA-based prospective longitudinal studies in women of childbearing age with mixed parity at baseline and including non-gravid comparison groups followed for up to10 years indicate that long-term maternal weight-gain is predominantly associated with the first birth with no further cumulative increases with additional births [27,28]. However we found no evidence to suggest that inter-pregnancy weight change was influenced by parity as irrespective of weight change cut-off (>10kg or >2 BMI units) there was no difference in the frequency or magnitude of gain or loss between the first and second, compared with the second and third pregnancy. This contrasts with a much earlier study of weight-change across parities in Aberdeen (n = 1325 women) whereby high weight-gains (defined as >7.5kg) were most common between the first and second pregnancy [20]. Notably the latter study was carried out in an era spanning post-war food rationing (1949–1964), and the magnitude of average weight-change between the first and third pregnancy, adjusted for age and year of delivery, was markedly lower than the current analysis of women living in the same geographical area (1.5kg vs. 5.1 and 6.0kg for women with a BMI<25 or >25, respectively at baseline, using the same adjustments). This emphasises the large secular increase in maternal weight in a representative UK obstetric population.

### Pregnancy complications, inter-pregnancy BMI-change and current BMI

The study demonstrates that inter-pregnancy weight-gain between the start of the first and second pregnancy modestly increases the risk of maternal morbidity at the second pregnancy and confirms previous analysis of this and other much larger populations [7,10,11,13]. In this instance a combined morbidity index incorporating pre-eclampsia, gestational hypertension, caesarean-delivery and thromboembolism was utilised as the study was underpowered to examine these pregnancy complications separately. Uniquely the analysis additionally demonstrates that maternal morbidity at the third pregnancy is influenced by weight-gain in the first as well as the second inter-pregnancy period but not by BMI-classification at the start of the current (third pregnancy). This implies that it is weight-gain across the preceding pregnancy and post-partum periods which confer risk. For hypertensive disease, weight-gain and the associated gradual increase in adipocyte mass and hence cytokine production leading up to the pregnancy of interest may progressively promote a subclinical inflammatory state. This together with other obesity related metabolic factors may impact early placental development and perfusion, increasing the risk of hypertension [29].

Herein weight-gain between the first and second pregnancy, and being overweight or obese by the third pregnancy protected the fetus against SGA-birth at the third delivery. This is broadly in line with previous observations involving larger numbers demonstrating that inter-pregnancy weight-gain was protective against both primary and recurrent SGA-birth at the second pregnancy, predominantly in women who had a healthy BMI at first pregnancy (n = 12740 and n = 24,520, respectively,[13,30]). Thus gaining weight across parities and reaching a higher BMI at the start of the third pregnancy was supportive of normal fetal growth. In addition to generally improving maternal nutrient reserves available for fetal growth at the start of the third pregnancy, the attenuated risk of SGA-birth may reflect an increase in placental size as we have previously shown that placental weight increases ~4.4g per BMI unit [31]. Accordingly when adjustment for placental weight at delivery was included in the model the protective effect of being obese by the third pregnancy was no longer statistically significant and the impact of being overweight was somewhat reduced (OR0.58, P<0.001 vs. OR0.67, P<0.05).

The present analysis demonstrates that maternal weight-loss in the inter-pregnancy period preceding the third pregnancy doubled the risk of SGA-birth at delivery. A similar association between inter-pregnancy weight-loss and increased risk of SGA-birth or low birthweight at the second delivery has variously been reported in women with a BMI below and above 25 units at first pregnancy [13,30,32,33]. Similarly, recent systematic appraisals examining the impact of weight-loss or positive weight-gain below IOM 2009 guidelines during pregnancy in obese women demonstrate that the incidence of LGA-birth can be reduced but at the expense of increased risk of SGA-birth [34,35]. While we were insufficiently powered to stratify the current population by baseline BMI-category, the analysis nevertheless supports and extends the robustness of the relationship between weight-loss and fetal growth-restriction across the childbearing period and up to the third pregnancy. We recognise that a newborn weighing ~2644g at the second or third delivery and defined as SGA using parity specific birth weight charts may have subtly different risks for morbidity than one weighing ~2543g at the first delivery (Table 1). However if we use birth weight centiles which are not parity specific the conclusions of the above analysis are unchanged. The contrasting risks associated with inter-pregnancy weight-change in opposite directions makes formulating dietary and lifestyle advice to optimise pregnancy outcome complex. While limiting excessive weight-gain before, during and between pregnancies is undoubtedly desirable to protect women from pregnancy-associated morbidity and longer term obesity, health professionals advising women need to be cognisant that weight-loss in the inter-pregnancy period may have a serious short term penalty in terms of increased risk of SGA-birth.

### Strengths and weaknesses

Strengths of the study include height and weight being measured by trained staff at the first antenatal appointment at a single maternity hospital for three consecutive pregnancies with weight adjusted to the same gestational age for all maternities: this also removes the recall bias associated with self-reported anthropometry. Further several important known confounders including age, year of delivery, inter-delivery interval and smoking habit were measured and the analysis adjusted accordingly. A potential weakness is the lack of information for weight-change in non-parous women to account for secular change in bodyweight and the inability to differentiate between weight-change during the index pregnancy versus post-partum. Women delivering in the UK are not consistently weighed in late pregnancy precluding gestational weight-change assessment, and testing for any association between gestational weight-change and maternal / fetal complications. The population size was wholly appropriate for the longitudinal assessment of weight-change across parities but relatively small for the assessment of the impact of weight-change on individual maternal complications and hence a combined maternal morbidity index was used. Nevertheless the individual pregnancy complication event rate in relation to parity status (primiparous/multiparous) agreed with previous publications using different populations [3–6, 7–12]. The data was collected over a period of time where changes in obstetrical practice may have occurred (eg. caesarean section criteria) but this possible bias was minimized by including year of delivery in the statistical models.

## Conclusion

Half the women studied exhibited significant weight fluctuations during three successive pregnancies. While women with a normal BMI at first pregnancy largely gain weight, those who are overweight or obese are at increased risk of substantial weight-gain and loss. Weight-gain during both inter-pregnancy periods increased the risk of maternal morbidity and as such weight-management interventions to limit gain may mitigate this risk irrespective of parity. However weight-gain between first and second pregnancy, and being overweight or obese by the third pregnancy protected the fetus against SGA-birth whereas weight-loss between the second and third pregnancy doubled the SGA risk. These contrasting effects of weight-change during the childbearing period may have implications for the long-term health of both mother and child.
